# Anti-Inflammatory Mechanisms of Curcumin and Its Metabolites in White Adipose Tissue and Cultured Adipocytes

**DOI:** 10.3390/nu16010070

**Published:** 2023-12-25

**Authors:** Tariful Islam, Shane Scoggin, Xiaoxia Gong, Masoud Zabet-Moghaddam, Nishan S. Kalupahana, Naima Moustaid-Moussa

**Affiliations:** 1 Department of Nutritional Sciences, Texas Tech University, Lubbock, TX 79409, USA; tariful.islam@emory.edu (T.I.); shane.scoggin@ttu.edu (S.S.); nkalupahana@uaeu.ac.ae (N.S.K.); 2 Obesity Research Institute, Texas Tech University, Lubbock, TX 79409, USA; mmoghaddam@absci.com; 3 Center for Biotechnology and Genomics, Texas Tech University, Lubbock, TX 79409, USA; xiaoxia.gong@bms.com; 4 Department of Nutrition and Health, College of Medicine and Health Sciences, United Arab Emirates University, Al Ain P.O. Box 15551, United Arab Emirates

**Keywords:** inflammation, obesity, curcumin, tetrahydrocurcumin, curcumin-O-glucuronide, white adipose tissue, high-fat diet, 3T3-L1 adipocytes, C57BL/6J (B6) mice

## Abstract

The plant-derived polyphenol curcumin alleviates the inflammatory and metabolic effects of obesity, in part, by reducing adipose tissue inflammation. We hypothesized that the benefits of curcumin supplementation on diet-induced obesity and systemic inflammation in mice occur through downregulation of white adipose tissue (WAT) inflammation. The hypothesis was tested in adipose tissue from high-fat diet-induced obese mice supplemented with or without curcumin and in 3T3-L1 adipocytes treated with or without curcumin. Male B6 mice were fed a high-fat diet (HFD, 45% kcal fat) with or without 0.4% (*w*/*w*) curcumin supplementation (HFC). Metabolic changes in these mice have been previously reported. Here, we determined the serum levels of the curcumin metabolites tetrahydrocurcumin (THC) and curcumin-O-glucuronide (COG) using mass spectrometry. Moreover, we determined interleukin 6 (IL-6) levels and proteomic changes in LPS-stimulated 3T3-L1 adipocytes treated with or without curcumin by using immunoassays and mass spectrometry, respectively, to gain further insight into any altered processes. We detected both curcumin metabolites, THC and COG, in serum samples from the curcumin-fed mice. Both curcumin and its metabolites reduced LPS-induced adipocyte IL-6 secretion and mRNA levels. Proteomic analyses indicated that curcumin upregulated EIF2 and mTOR signaling pathways. Overall, curcumin exerted anti-inflammatory effects in adipocytes, in part by reducing IL-6, and these effects may be linked to the upregulation of the mTOR signaling pathway, warranting additional mechanistic studies on the effects of curcumin and its metabolites on metabolic health.

## 1. Introduction

Obesity is a complex metabolic disease that is characterized by chronic low-grade inflammation [1] and the excessive expansion of white adipose tissue (WAT) [2]. Currently, two out of every five American adults suffer from obesity. By the year 2030, it is predicted that almost half of US adults might be suffering from this complex metabolic disease [3]. Together with the increasing number of people with obesity, treatment costs are also rising in parallel; compared with a person without obesity, a person with obesity spends at least USD 600 more, each year, on out- or inpatient visits as well as prescription drug costs [4]. In general, medical costs may be 30% higher for a person suffering from obesity than a healthy-weight person [5]. While the etiology of obesity is multifactorial, dietary factors are important contributors to obesity [6,7]. For instance, a Western diet that is high in calories, fat, and sugar content exacerbates obesity and further increases the risk of other comorbid conditions such as type two diabetes mellitus (T2D), inflammatory disorders, and heart disease [8,9]. Therefore, it is critical to address the obesity epidemic by using diverse, safe, and cost-effective preventive and treatment strategies.

Adipose tissue as an endocrine organ was first suggested as a major contributor to reproductive tract cancer in women with obesity [10]. Later, it was found that adipose tissue secretes different types of “adipocytokines” including inflammatory interleukin-6 (IL-6) [11,12] which may link obesity to other metabolic and chronic diseases. Free fatty acids and bacterial endotoxin lipopolysaccharide (LPS) are other key elements that contribute to obesity-associated inflammation via increased inflammatory adipokines secretion from adipose tissue [13,14], which in turn interfere with insulin signaling to promote insulin resistance [15,16].

Natural food components have long been recognized for their protective effects against many chronic diseases; however, evidence-based research for many of these compounds is lacking. Polyphenolic compounds exert anti-obesity properties in many preclinical and some clinical trials [17]. Among these, curcumin, a food spice found in the *Curcuma longa* root which has traditionally been used in parts of China and Southeast Asia, is claimed to be an anti-diabetic, anti-inflammatory, and anti-obesity agent [17,18]. Previously, we reported that curcumin exerted protective metabolic effects in diet-induced obese mice, independent of changes in body weight [3]. Curcumin reduced the total macrophage infiltration and adiposity of the WAT along with downregulating genes involved in the inflammatory Tlr-4/NfkB/Stat1 pathway in the B6 mouse model. However, the mechanisms underlying these effects of curcumin have not been completely unraveled. Despite encouraging results for curcumin’s effects on obesity and associated metabolic dysfunctions [19,20], its bioavailability is a major concern [21,22]. Recent findings suggest that curcumin metabolites, such as tetrahydrocurcumin (THC) and curcumin–O–glucuronide (COG), may also have anti-obesity effects [3,23], thus redefining the role of curcumin metabolism in light of its low bioavailability [24].

In addition, to further determine the direct effects of curcumin in adipocytes, we performed proteomic analyses in differentiated 3T3-L1 cells stimulated with LPS to induce inflammation and then treated them with or without curcumin. The proteomics data of adipocytes suggest that EIF2 and mTOR signaling pathways are upregulated by curcumin. Cell culture studies further confirmed that the anti-inflammatory effects of the curcumin metabolites THC and COG, which are similar to curcumin, reduced LPS-induced IL-6 secretion in differentiated adipocytes. 

## 2. Materials and Methods

### 2.1. Animal Studies

Details of the animal study, including curcumin dosage details, and the animal diet used in this project were reported previously by our group [3]. Briefly, five-week-old male B6 mice (Jackson Laboratory, Bar Harbor, ME, USA) were single-housed in specific-pathogen-free (SPF) cages at 23 °C. The animal study protocol was approved by the Texas Tech University Institutional Animal Care and Use Committee (TTU protocol numbers16011-04 and 19034-04). The mice were separated into two groups: HF and HFC (*n* = 8–10 per group). The food composition for both groups contained 45% kcal fat, 35% carbohydrates, and 20% protein. However, only the HFC group’s food was prepared with 0.4% (*w*/*w*) curcumin (98.24% pure) (Santa Cruz Biotechnology Inc., Dallas, TX, USA). After 14 weeks of feeding, the mice were fasted for 5 h before euthanasia by CO_2_ asphyxiation. Their blood was collected using cardiac puncture followed by serum collection using polymer gel containing BD microtainers (Becton, Dickinson & Co, Franklin Lakes, NJ, USA). The collected tissue samples were flash-frozen in liquid nitrogen. The serum and snap-frozen samples were stored at −80 °C until analysis.

### 2.2. RNA Extraction, cDNA Preparation, and Quantitative Real-Time PCR

The procedures used were as previously described [3]. Briefly, WAT was homogenized in QIAzol lysis reagent (Qiagen, Germantown, MD, USA) using a tissue homogenizer (TissueLyser LT, Qiagen, Germantown, MD, USA). After chloroform separation, total RNA was isolated using Quick-RNA kits (Zymo Research, Irvine, CA, USA), and RNA concentrations were measured using Take3 (Biotek Cytation 3, Winooski, VT, USA). For cDNA synthesis, Maxima H Minus cDNA Synthesis Master Mix (ThermoFisher Scientific, Waltham, MA, USA) and BioRad T100 thermal cycler (BioRad, Hercules, CA, USA) were used.

Using the quantitative polymerase chain reaction (qPCR), gene expression was performed, whereby PowerUp SYBR Green Mastermix (ThermoFisher Scientific, Waltham, MA, USA) and the QuantaStudio 3 device (ThermoFisher Scientific, Waltham, MA, USA) were used. The primers were purchased from MilliporeSigma (St. Louis, MO, USA) and relative gene expression was calculated using QuantStudio Design & Analysis cloud application (ThermoFisher Scientific, Waltham, MA, USA).

### 2.3. Measurement of THC and COG in Curcumin, Serum, and Adipocytes

Quantification and detection of THC and COG in curcumin, serum, and adipocytes was conducted using liquid chromatography–mass spectrometry (LC-MS/MS), which was also detailed previously [3]. While preparing the calibration curve, for THC quantification, a 5 µL aliquot of the standard solutions of THC (Millipore Sigma, St. Louis, MO, USA) and 5 µL hesperetin (internal standard, 10 µg mL^−1^) (Millipore Sigma, St. Louis, MO, USA) were added into 40 µL plasma to a final concentration of 1, 2, 5, 10, 50, 200, 500, and 1000 ng mL^−1^. THC extraction from the serum was conducted as described previously. After a gentle vortex and dilution with 200 µL phosphate buffer saline (PBS, 0.1 M, pH 3.2), 1 mL ethyl acetate was added for extraction through vortex mixing. After centrifugation at 13,500 rpm for 4 min, the supernatant was collected and dried and then reconstituted in 50 µL of mobile phase. After centrifugation, a 25 µL aliquot of the supernatant was injected for LC-MS/MS analysis. THC and hesperetin chromatographic separation were performed by using a Vanquish ultra-high performance liquid chromatography (UHPLC) system (ThermoFisher Scientific, Waltham, MA, USA). An Acquity BEH C8 column (2.1 mm × 100 mm, 1.7 µm, 130 A, Waters, Milford, MA, USA) was applied using a 10 min isocratic gradient (60% solvent B) at a flow rate of 0.2 mL min^−1^ with solvent A (water and 0.1% formic acid) and solvent B (acetonitrile and 0.1% formic acid). MS analysis was performed using a Q-Exactive HF (Thermo Fisher Scientific, Waltham, MA, USA) mass spectrometer in negative ion mode. A parallel reaction monitoring (PRM) method was developed with the full-scan MS (*m*/*z* 150–2000) and HCD MS/MS scan using NCE at 25 for THC and NCE at 45 for hesperetin. The obtained calibration curve mentioned above was used for serum THC quantification. Similarly, we also prepared the calibration curve for COG (Synthose Inc., Concord, ON, Canada), which is detailed elsewhere [3].

### 2.4. Cell Culture Studies

We used our previously reported cell culture procedure with minor modifications [25]. Briefly, 3T3-L1 preadipocytes were cultured in a humidified atmosphere of 5% CO_2_, 95% air at 37 °C using six-well plates, in growth media containing Dulbecco’s modified Eagle’s medium (DMEM) (Thermo Fisher, Pittsburg, PA, USA) supplemented with antibiotics consisting of 1% penicillin–neomycin–streptomycin (Thermo Fisher, Pittsburg, PA, USA) and 10% fetal bovine serum (FBS) (Atlas Biologicals, Fort Collins, CO, USA). When the cells reached ~95% confluence, they were switched to the differentiation media for 2–3 days (DMEM with 0.5 mM of 1-methyl-3-isobutylxanthine, MIX; 0.25 µM of dexamethasone, DEX; and 10 ng/mL of insulin (Millipore Sigma, St. Louis, MO, USA)). After differentiation was induced as described above, the cells were kept in growth media supplemented with insulin for two additional days to enhance lipid accumulation in the cells and then maintained in growth media for up to a week, until maximum differentiation occurred, with the media changed every other day.

We assessed 3T3-L1 adipocyte viability with curcumin (Millipore Sigma, St. Louis, MO, USA) and its metabolites, COG (Synthose Inc., ON, Canada) and THC (Millipore Sigma, St. Louis, MO, USA), using different concentrations (between 1 µM–15 µM) with *n* = 4–6 wells per treatment group. Viability was assessed using the MTT assay, as described below. The cells were treated with 5 mg/mL of Thiazolyl Blue Tetrazolium Bromide (Millipore Sigma, St. Louis, MO, USA) for 4 h and then 1 mL of DMSO used as stop solution (Fisher Scientific, Hampton, NH, USA). Absorbance of the homogenized cells was measured using a Biotek Cytation 3 multiplate reader (Winooski, VT, USA) at 570 nm with background subtraction from readings at 690 nm.

### 2.5. 3T3-L1 Cell Culture Treatments

3T3-L1 preadipocytes and adipocytes were treated for 18 to 20 h with 200 ng/mL of lipopolysaccharide (LPS) (Millipore Sigma, St. Louis, MO, USA) with or without 5 µM curcumin. LPS was used to induce adipocyte inflammation with an additional unstimulated and untreated control group labeled as LPS−. The cells that were treated with LPS and curcumin or LPS alone were labeled LPS+Cur and LPS+, respectively.

### 2.6. Oil Red O Staining (Lipid Staining) of Curcumin-Treated Adipocytes

For Oil Red O staining, the cells were grown as described in Section 2.5. Curcumin was added from day 1 of differentiation until the cells were fully differentiated. Differentiated cultured adipocytes were washed with phosphate-buffered saline and then fixed with 10% formalin and stained with Oil Red O solution following the manufacturer’s protocol (O0625, Millipore Sigma, St. Louis, MO, USA). Once the Oil Red O solution was removed, the cells were washed 3 times with deionized water and then imaged using a microscope (Invitrogen, Carlsbad, CA, USA). Oil Red O staining of the adipocytes was eluted into isopropanol (200 µL/well of a 24-well plate) and quantified by reading the absorbance at 540, which is proportional to the lipid content in the cells.

### 2.7. ELISA Cytokine Assays

Following the cell treatments, media were collected to analyze the levels of secreted pro-inflammatory adipokines (IL-6 and MCP-1). We used ELISA cytokine assay procedures according to the instructions of the manufacturer (R&D Systems, Minneapolis, MN, USA). Serial dilutions of standards of IL-6 and MCP-1 were used to create the standard curves.

### 2.8. Protein Analysis by Mass Spectrometry

#### 2.8.1. Protein Extraction, Tryptic Digestion, and Analysis by LC-MS/MS

3T3-L1 cells were treated with 200 ng/mL of LPS with either vehicle (Cur-) or 1 µM curcumin. The cells were harvested, and cell proteins were extracted using a 5% sodium deoxycholate (SDC) buffer, in 100 mM ammonium bicarbonate (ABC) buffer. Then, equal amounts of beads (400 µm of zirconium beads) were added to each tube and homogenized in the cold, and then, the samples were centrifuged at 15,000 rpm at 4 °C for 15 min. The supernatant containing the proteins was collected and stored at −80 °C until further protein analyses.

BCA (bicinchoninic acid) assay was used to quantify the total amount of proteins in the WAT samples, using bovine serum albumin (BSA) as a standard. The absorbance of all samples was measured with a spectrophotometer (Synergy H1 Microplate reader (Biotek)) at 562 nm for 10 min.

Approximately 50 µg of protein was mixed with ABC buffer to make a 250 µL final solution. Subsequently, the protein samples were reduced, alkylated, and digested by trypsin overnight, and the resulting peptides were analyzed by LC-MS/MS.

#### 2.8.2. Protein Identification, Label-Free Quantification, and Data Analyses

The raw MS/MS data from all samples were imported into MaxQuant (v1.6.14), a computational proteomics platform, and label-free quantification (LFQ) of the identified proteins was performed. The proteins were identified using *Mus musculus* protein reference proteomes (uniport-mouse-filtered-reviewed_yes) downloaded from UniProt and imported into MaxQuant. MaxQuant identified and quantified the proteins in the samples based on calculating the area on the peak. To obtain quantitative data for all peptides in the samples, peak intensities from the whole set of measurements were compared.

Perseus version v1.6.14.0, freely available software, was used to perform the statistical analysis wherein the LFQ protein intensities from the MaxQuant analysis were imported and transformed. Data filtering was carried out to remove unnecessary or incorrect protein identifications from the main data frame. Two-sample tests were applied to determine if the means of the LFQ intensity values of the two categories of samples were significantly different from each other. The threshold *p*-value was considered to be 0.05 (5% probability error).

#### 2.8.3. Pathway and Network Analyses

Once the differentially expressed proteins were determined, we further evaluated the enriched pathways and protein functions and identified enriched canonical pathways using ingenuity pathway analysis (IPA^®^) software (v6.0, QIAGEN, Redwood City, CA, USA). The analyzed data were visualized using the ClustVis web tool [26] and GraphPad Prism Software 8.2 (GraphPad, San Diego, CA, USA).

### 2.9. Statistical Analyses

For the statistical analysis, GraphPad Prism Software 8.2 (GraphPad, San Diego, CA, USA) was used. Data obtained from the HF and HFC mice groups were analyzed with unpaired *t*-tests. The differences were considered significant at a *p*-value < 0.05. For the cell culture data, one-way ANOVA was applied with Tukey’s post hoc test. We used Student’s *t*-test for proteomic data analysis. All data are presented as the mean ± SEM.

## 3. Results

### 3.1. Mouse Data

Details of the mouse study data, including but not limited to food composition, body weight, fat weight, food consumption, and oxygen consumption, are reported elsewhere [3].

#### Curcumin Metabolites Tetrahydrocurcumin (THC) and Curcumin-O-Glucuronide (COG) in Serum

**Figure 1:**
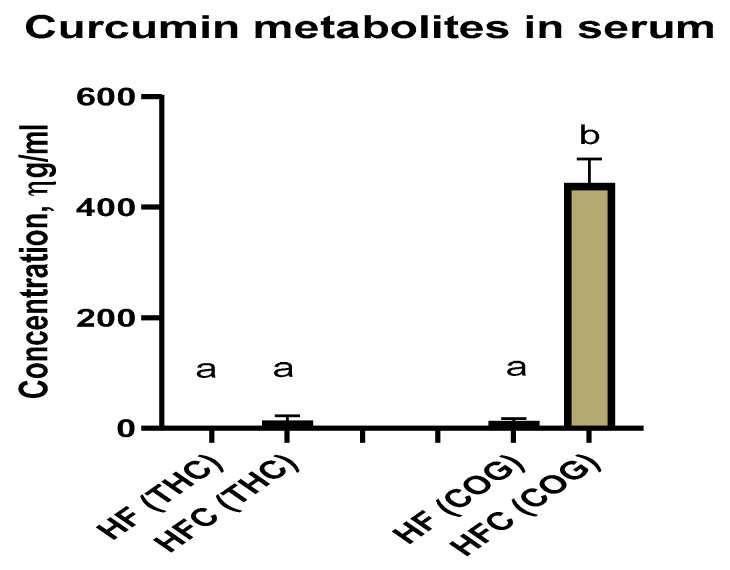
Detection of curcumin metabolites in mouse serum. We used the mass spectrometry technique to detect curcumin metabolites in the serum samples of the HF and HFC group mice. Tetrahydrocurcumin (THC) and Curcumin-O-glucuronide (COG) were detected in the serum samples of the HFC-fed mice. Means without a common letter differ (*p*-value < 0.05), *n* = 3.

THC and COG were detected in the serum samples of the HF and HFC groups (Figure 1). However, curcumin was not detected in the serum samples.

### 3.2. Adipose Cell Culture

#### 3.2.1. Cell Viability and Dose–Response Studies

**Figure 2:**
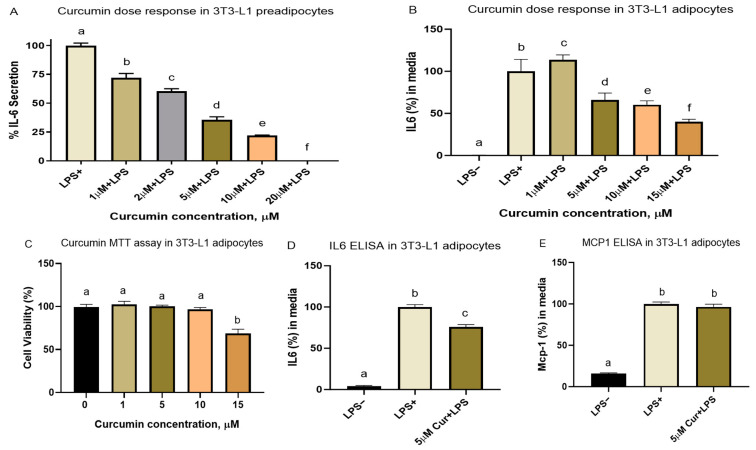
Curcumin dose–response and cell viability experiments: Curcumin reduced LPS-induced inflammatory cytokine IL-6 secretion in the cultured adipocytes. (**A**) In the non-differentiated preadipocytes, curcumin dose-dependently reduced IL-6 secretion at all doses (1–20 µM). (**B**) However, in the differentiated adipocytes, curcumin doses between 5–15 µM were more effective in reducing IL-6 secretion induced by LPS (200 ng/mL). (**C**) Up to 10 µM, curcumin did not induce any cell toxicity. In the differentiated adipocytes, a 5 µM dose of curcumin was the lowest dose that reduced IL-6 secretion without any toxicity and had similar effects compared to 10 µM. Thus, this dose was applied in the subsequent cell culture experiments. The MTT assay results were averaged from 2 biological replicate experiments, where 4 to 5 wells per treatment were used for each treatment group per experiment. (**D**,**E**) A curcumin dose of 5 µM reduced IL-6 secretion in LPS (200 ng/mL)-induced mature 3T3-L1 cells. However, MCP-1 secretion was not affected by curcumin at the dose used. Means without a common letter differ (*p*-value < 0.05), *n* = 3; 4–6 wells per treatment group in each experiment.

The cell viability test indicated that, up to 10 µM, curcumin effects were not toxic to the differentiated adipocytes. We conducted dose–response experiments in 3T3-L1 pre-adipocytes using curcumin obtained from Santa Cruz, CA, USA (≥98.4% curcumin). There, we observed that 1 µM of curcumin is the lowest concentration that can reduce 200 ng/mL of LPS-induced inflammation in pre-adipocytes. Later, we had to purchase different lots (HPLC grade, purity ≥ 80% curcumin) from Millipore Sigma, (St. Louis, MO, USA). The second dose–response and cell viability tests in the differentiated adipocytes confirmed that a 5 µM dose of curcumin is the lowest concentration that reduced inflammation in the differentiated 3T3-L1 cells without any toxic effects. Both 10 and 15 µM doses of curcumin also reduced inflammation in the differentiated 3T3-L1 adipocytes (Figure 2). However, as per the cell viability test (MTT) results, 15 µM of curcumin also reduced cell viability. Therefore, we used a 1–5 µM curcumin dose for all subsequent cell culture treatments with curcumin.

The curcumin dose of 5 µM reduced LPS-induced inflammation in the LPS-stimulated adipocytes. Curcumin reduced the media IL-6 levels (but not MCP-1 at the dose tested) by 24% compared to the adipocytes treated with LPS only (Figure 2).

#### 3.2.2. Effect of Curcumin on Lipid Accumulation during Adipocyte Differentiation

The cells were treated with curcumin after full differentiation into adipocytes (Supplementary Figure S1A) or during differentiation (Supplementary Figure S1B). No significant effects of curcumin on lipid accumulation in adipocytes were observed in both experiments, except for the 15 µM dose, for which we observed reduced cell viability (Supplementary Figure S1C).

#### 3.2.3. Curcumin Metabolites THC and COG Reduced IL-6 Secretion in LPS (200 ng/mL)-Induced Differentiated 3T3-L1 Adipocytes

**Figure 3:**
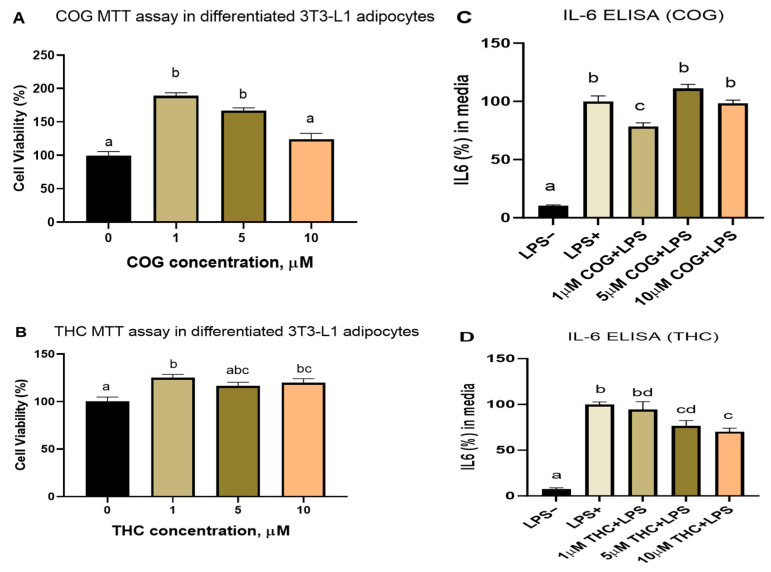
Dose–response effects of curcumin metabolites (COG and THC) on cell viability and IL-6 secretion. (**A**,**B**) The MTT assay showed that none of the doses (1–10 µM) of curcumin metabolites tested reduced cell viability in the 3T3-L1 adipocytes. (**C**) In the differentiated adipocytes, only 1 µM of COG reduced IL 6 secretion; (**D**) THC dose-dependently reduced IL-6 secretion with significant effects at 5 and 10 µM of THC. Means without a common letter differ (*p*-value < 0.05); duplicate experiments with 6–12 wells in each MTT test and 4–6 wells in each ELISA test were carried out.

We first tested the cell viability of the differentiated adipocytes treated with different doses (1 µM, 5 µM, and 10 µM) of curcumin metabolites (THC and COG) but detected no toxic effects (Figure 3A,B).

However, 1 µM of COG but no other doses reduced IL-6 secretion in the differentiated 3T3-L1 adipocytes. On the other hand, THC dose-dependently reduced LPS-induced inflammation in the 3T3-L1 adipocytes.

#### 3.2.4. Detection of THC and COG in Curcumin-Treated Adipocytes

When we analyzed the raw curcumin powder, COG but not THC was detected there (Table 1 and Supplementary Figure S2). None of these metabolites were detected in the control group (no LPS- or curcumin-treated adipocytes), while we detected THC in the LPS+Curcumin-treated adipocytes.

#### 3.2.5. Proteomic Analyses of Curcumin-Treated Adipocytes

**Figure 4:**
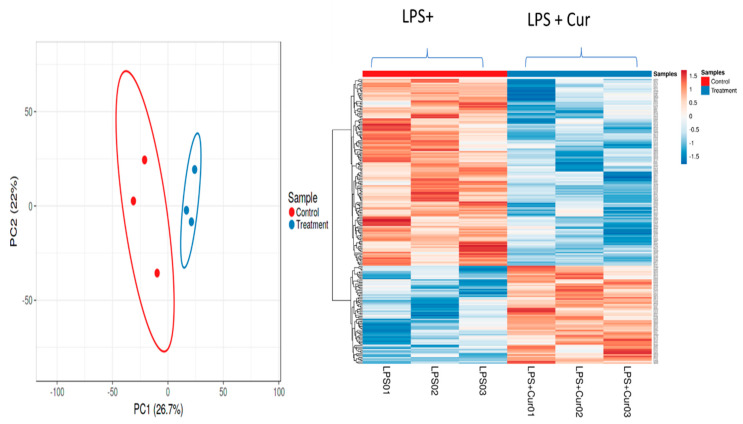
Proteomic analyses of curcumin effects in 3T3-L1 adipocytes. A total of 2106 proteins were differentially expressed, of which 209 proteins were significantly up- or downregulated by curcumin (0.2-fold change cut-off; *n* = 3, control = 200 ng/mL of LPS and treatment = 200 ng/mL of LPS + 1 µM curcumin).

We conducted mass spectrometry analysis of protein extracts from the LPS-stimulated adipocytes treated with or without 1 µM of curcumin. A total of 2106 differentially expressed proteins were identified in these cells, of which 209 proteins were significantly and differentially expressed between the curcumin and non-curcumin-treated cells (Figure 4). A threshold value of 0.2-fold significant changes was used in the IPA pathway analysis application (IPA, QIAGEN, Redwood City, CA, USA), which yielded 98 significantly regulated proteins above the selected threshold value. These proteins were mapped to various canonical signaling pathways. Based on the *p*-values, we present the top eight canonical signaling pathways in Table 2, where a positive z-score means activation and a negative z-score means downregulation of the respective signaling pathways. The ratio was obtained by dividing the number of differentially expressed genes in the canonical signaling pathways by the total number of genes in the pathways.

**Figure 5:**
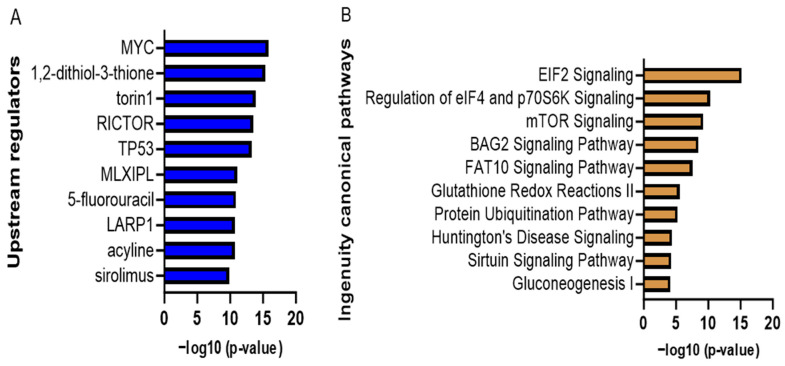
Curcumin-modulated upstream regulators (**A**) and pathways (**B**) (ranked based on statistical significance). The adipocytes’ proteomics data were analyzed with IPA pathway analysis application. The EIF2 and mTOR pathways were upregulated (z-score: 3 and 1.342, respectively, *p*-value < 0.05), which was supported by the inhibition of the upstream regulator of Rictor and Sirolimus (z-score of −2.132 and −2.693, respectively, *p*-value < 0.05). GraphPad software was used for this visualization.

The differentially expressed metabolic and signaling pathway analysis revealed that EIF2 and mTOR signaling appear in the top three upregulated pathways (Figure 5). The upstream regulator protein analysis revealed that mTOR signaling associated with RICTOR and Sirolimus is on the list. RICTOR is a binding protein of mTOR signaling that assists in the mTOR signaling pathway. Sirolimus is a kinase inhibitor that inhibits mTOR kinase.

## 4. Discussion

Obesity is an alarming public health issue in the USA. Preventing obesity and associated complications with curcumin has promising prospects. However, details on the molecular mechanisms of the curcumin-mediated alleviation of diet-induced obesity-associated inflammation is still obscure.

Curcumin increases the activity of mTOR signaling in the WAT of B6 mice [27]. Increased mTOR activity can induce insulin resistance [28,29]. Although we observed increased mTOR activity in the curcumin-treated mature adipocytes, the mouse study did not show any sign of insulin resistance development [3]. In line with this, mTOR gene expression was not upregulated in the WAT in the curcumin-fed mice (Supplementary Figure S3), suggesting neutral effects of curcumin on WAT mTOR gene expression. In addition, *Ampk* and *Irs1* gene expression levels were unchanged, suggesting that the effects of curcumin were insulin-independent (Supplementary Figure S3). On the other hand, the mouse experiment showed that the lack of response of mTOR genes to curcumin may indicate that it is likely regulated at the post-translational level, which we have not examined yet. Therefore, more in-depth studies focusing on post-translational modifications should confirm the interactions between curcumin and the mTOR signaling pathway in WAT.

We also observed that curcumin downregulated LPS-induced IL-6 secretion in 3T3-L1 adipocytes but not MCP-1 secretion. MCP-1 is an important inflammatory protein that is associated with the pathogenesis of insulin resistance [30]. It has been reported that free fatty acids (FFAs), a Tlr-4 agonist, and TNF-α combinedly create a superior environment to trigger inflammatory cytokine secretion during obesity, which is mediated by the TLR4/TRIF/IRF3 signaling cascade [31], and LPS (100 ng/mL) is a potent stimulator of IL-6 secretion in 3T3-L1 cells via the Tlr-4/NfkB pathway [32]. Curcumin’s ability to reduce LPS-induced IL-6 secretion implies that curcumin targets the Tlr-4/NfkB/IL-6 inflammatory pathway. Interestingly, like immune cells, adipocytes also secrete cytokines, including IL-6 and MCP-1 [33]. LPS (200 ng/mL) also stimulates cytokine secretion, including IL-6 and MCP-1, from 3T3-L1 adipocytes, independent of immune cells such as macrophages [33]. Our results suggest that curcumin targets specific inflammatory pathways in adipocytes that downregulate the secretion of IL-6 but not MCP-1. It is possible that different treatment times or doses are needed for 3T3-L1 adipocyte responses to curcumin. However, this observation contradicts a previous report wherein 10 µM of curcumin suppressed MCP-1 secretion in 3T3-L1 adipocytes when inflammation was induced by mesenteric adipose tissue-conditioned medium [34]. It is possible that this discrepancy is in part related to the different cell types used (primary adipose tissue in this reference vs. 3T3-L1 adipocytes in our current study). Another study confirmed that curcumin (10 µM) can reduce IL-6 secretion from 1 µg/mL LPS-treated 3T3-L1 adipocytes [35]. Interestingly, curcumin also reduced palmitate (a Tlr-4 agonist like LPS)-induced IL-6 secretion from 3T3-L1 adipocytes [36]. These findings indicate that curcumin targets the Tlr-4/NfkB/IL-6 inflammatory pathway in adipocytes while reducing inflammation. This observation is consistent with previously reported findings that curcumin reduces WAT inflammation, stimulated by HFD in B6 mice, via the Tlr-4/NfkB pathway in adipose tissue [3,37].

In epididymal white adipose tissue, curcumin upregulated eukaryotic translation initiation factor 2 (eIF2) signaling by suppressing eIF2α phosphorylation in the B6 mice model [27]. These observations were also associated with reduced serum glucose levels. Likewise, dephosphorylation of eIF2 was reported to enhance glucose tolerance in transgenic integrated stress response (ISR)-defective mice [38]. We and others have reported that curcumin increases glucose tolerance in B6 mice [3,39]. In this study, 3T3-L1 cell culture analysis revealed that curcumin’s effect on differential protein expression increases eIF2 signaling, supporting the role of eIF2 signaling in curcumin mediating enhanced glucose tolerance.

However, a major issue regarding the protective roles of curcumin in obesity-associated complications or other diseases is its low bioavailability. Interestingly, curcumin metabolites may potentially explain the effectiveness of curcumin (via conversion to these metabolites) despite its low bioavailability. In this study, we reported the detection of the curcumin metabolites tetrahydrocurcumin (THC) and curcumin-O-glucuronide (COG) in the serum samples from the curcumin-treated mice (while curcumin itself was not detected). Moreover, we detected THC in curcumin-treated differentiated adipocytes, although the raw curcumin powder was free from THC (Supplementary Figure S2), indicating that THC was a product of curcumin metabolism and likely one of the mediators of curcumin’s effects. Additionally, THC- and COG-treated cultured adipocytes demonstrated the potent effects of these metabolites in reducing LPS-induced inflammatory cytokine IL-6 secretion (Figure 3). Nevertheless, further pharmacokinetic analyses of curcumin and its metabolomic profiles in serum and tissues are warranted to better understand curcumin’s bioavailability and metabolic effects. Additional mechanistic studies are also required to dissect specifically how curcumin metabolism in adipocytes mediates curcumin/its metabolites effects on obesity. It is also important to highlight the important role of the metabolism of curcumin and other polyphenols by gut bacteria in their protective effects on metabolic diseases.

When we treated the 3T3-L1 cells with curcumin metabolites, they also responded in a similar manner to curcumin. We acknowledge this as a limitation of our current research, which merits further confirmation. Curcumin also downregulated the lipid content accumulation in adipocytes (Supplementary Figure S1) when the cells were treated during differentiation, indicating that curcumin reduced adipogenesis at 15 µM, which is consistent with reduced *Ppar-γ* expression by curcumin, in adipose tissue [3]. However, these findings must be interpreted with caution, as 15 µM of curcumin also reduced cell viability (Figure 2).

This study has some limitations to be addressed in future studies. First, we only used male B6 mice and not females. We initially used B6 males because they developed more robust inflammation under diet-induced obesity conditions, and our interest was to determine whether curcumin would reduce inflammation. However, it is still worthwhile to explore the other metabolic benefits of curcumin in females. This is because our previous data and those of others [40] showed that in B6 mice, males were more prone to developing systemic and WAT inflammation in response to DIO. However, it will be worth testing the effects of curcumin in females in future studies to determine other potential sex-related differences in response to curcumin. Next, we were focused on adipose tissue and adipocytes only. We acknowledge that the effects of curcumin in other tissues, such as those of the liver, brain, and muscle, are equally critical to understand. Likewise, we did not investigate the effects of curcumin metabolites in adipocytes using proteomics/mass spectrometry techniques.

## 5. Conclusions

Overall, our in vivo and in vitro studies demonstrate that curcumin alleviated diet-induced obesity-associated inflammation. Our proteomic data suggest that these effects may be mediated by the mTOR pathway. A recent study reported that mTOR inhibition enhanced LPS-mediated IL-6 secretion in monocytes [41]. Thus, it is possible that the effects of curcumin and its metabolites on the suppression of LPS-mediated IL-6 secretion is mediated by mTOR activation. Given the increased bioavailability of curcumin metabolites (THC and COG) in the curcumin-fed mice, and the direct effects of these metabolites on the cultured adipocytes, especially for THC, curcumin’s effects may have been mediated through its metabolic products, which are produced in the gut, liver, or other tissues. Overall, we observed limited changes in the gene expression of inflammatory and metabolic markers in response to curcumin, in cultured adipocytes, compared to previously reported effects of curcumin in mouse adipose tissue. However, since many of these genes are post-translationally regulated, and may be targeted by curcumin metabolites, rather than curcumin itself, we need to perform additional studies to confirm these hypotheses and determine the mechanisms underlying the effects of curcumin or the direct effects of its metabolites in adipose tissue and obesity. These future experiments, to be conducted both in vitro and in vivo, will provide critical insight into the mechanisms of curcumin in obesity and the potential use of its metabolites in preventing/treating metabolic diseases.

## Figures and Tables

**Table 1:** Curcumin metabolite detection through use of mass spectrometry.

Samples	THC Detection	COG Detection
Control (no LPS or curcumin)	No	No
LPS + curcumin-treated adipocytes	Yes	Not analyzed
Curcumin powder	No	Yes

**Table 2:** Curcumin increased EIF2 and mTOR signaling pathways.

Ingenuity Canonical Pathways	−log (*p*-Value)	Ratio	z-Score
EIF2 Signaling	15.2	0.0938	3
Regulation of eIF4 and p70S6K Signaling	10.3	0.0838	-
mTOR Signaling	9.25	0.0708	1.342
BAG2 Signaling Pathway	8.52	0.119	-
FAT10 Signaling Pathway	7.56	0.143	-
Glutathione Redox Reactions II	5.6	0.75	-
Protein Ubiquitination Pathway	5.27	0.0436	-
Sirtuin Signaling Pathway	4.29	0.0377	0.816

## Data Availability

Metadata and data described and analyzed in the manuscript will be made available after publication by the corresponding author.

## Supplementary Materials

The following supporting information can be downloaded at: https://www.mdpi.com/article/10.3390/nu16010070/s1, Figure S1: Effects of curcumin on lipid accumulation in 3T3-L1 adipocytes; Figure S2: Extracted ion chromatograms of THC, COG and Curcumin from the sample of curcumin powder, by LC-MS/MS; Figure S3: Curcumin did not influence insulin signaling related gene expression in mice WAT.

## List of Abbreviations

ABC	Ammonium bicarbonate	mTOR	Mammalian target of rapamycin
Ampk	AMP-activated protein kinase	NfkB	Nuclear factor kappa-light-chain-enhancer of activated B cells
COG	Curcumin-o-glucuronide	LPS	Lipopolysaccharide
DIO	Diet-induced obesity	LBP	LPS-binding protein
eIF2	Eukaryotic translation initiation factor 2	NRF1	Nuclear respiratory factor 1
FFA	Free fatty acid	Ppar-γ	Peroxisome proliferator-activated receptor gamma
HFD	High-fat diet	Stat1	Signal transducer and activator of transcription 1
HFC	High-fat diet with curcumin	Sirt1	Sirtuin 1
IkB	Inhibitory kappa-B		
IL-6	Interleukin-6	THC	Tetrahydrocurcumin
IPA	Ingenuity pathway analysis	Tlr4	Toll-like receptor-4
IRF3	interferon regulatory factor 3	TMP	Transmembrane proteins
Irs1	Insulin receptor substrate 1	T2D	Type 2 diabetes
ISR	Integrated stress response	TNF-α	Tumor necrosis factor-alpha
LFQ	Label-free quantification	TRIF	Toll/IL-1R domain-containing adaptor-inducing IFN-β
Mcp1	Monocyte chemoattractant protein-1	WAT	White adipose tissue
