# Supplementary material for: Anti-Inflammatory Mechanisms of Curcumin and Its Metabolites in White Adipose Tissue and Cultured Adipocytes
Source: Nutrients. 2023 Dec 25;16(1):70. doi: 10.3390/nu16010070 (PMC10780365; doi:10.3390/nu16010070)
Supplement: Supplementary file 1 [file nutrients-16-00070-s001.zip › nutrients-2758257-supplementary.pdf]

Anti-Inflammatory Mechanisms of Curcumin in White Adipose Tissue of Diet-Induced Obese Male Mice

Islam et al, Nutrients.

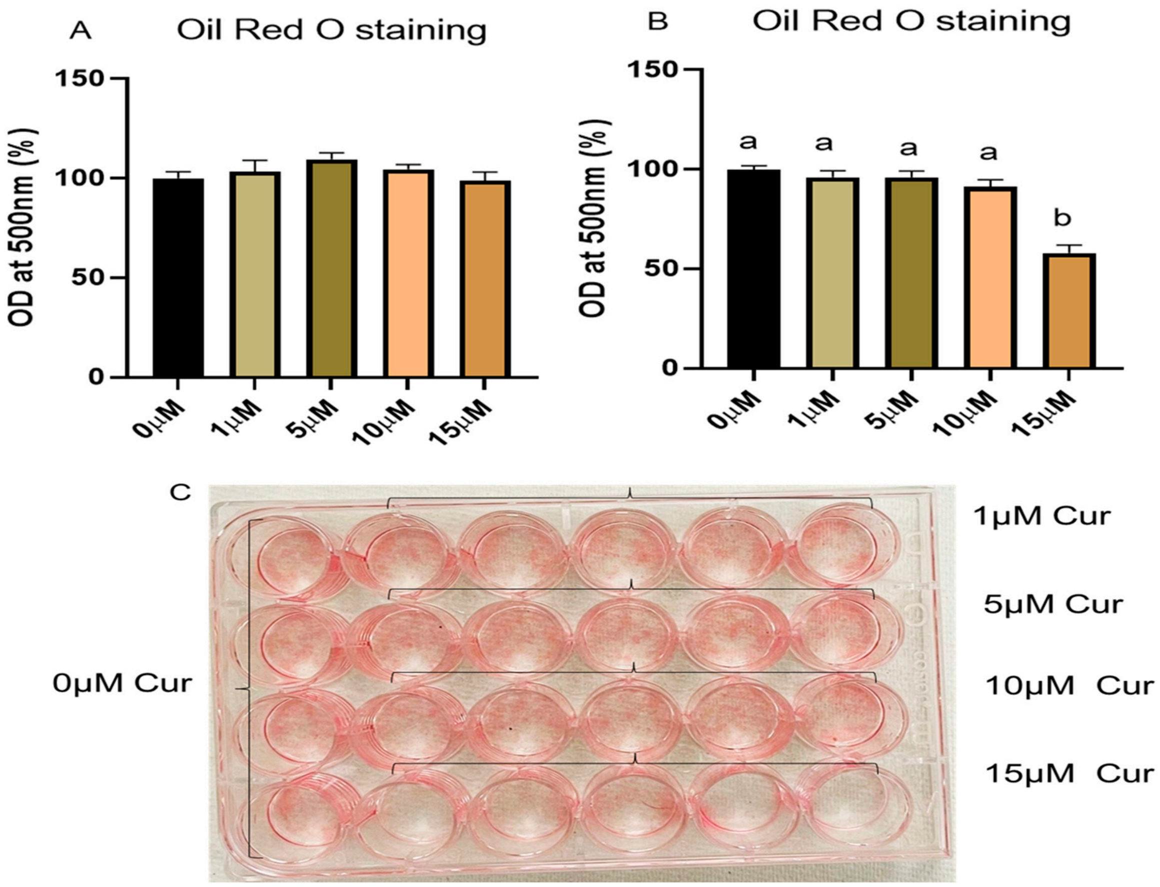

Supplementary Figure 1. Effects of curcumin on lipid accumulation in 3T3-L1 adipocytes. Oil red O staining was applied to 3T3-L1 adipocytes treated with different doses of curcumin (0 – 15 μM): A: post-differentiation; or B, C: during differentiation. Only 15 μM dose treatment during differentiation reduced lipids, which may be in part due to fewer cells according to Figure S1 C. Means without a common letter differ (*p*-value<0.05), *N* = 2 biological experiments, with 4-5 wells per treatment in each experiment.

Anti-Inflammatory Mechanisms of Curcumin in White Adipose Tissue of Diet-Induced Obese Male Mice

Islam et al, Nutrients.

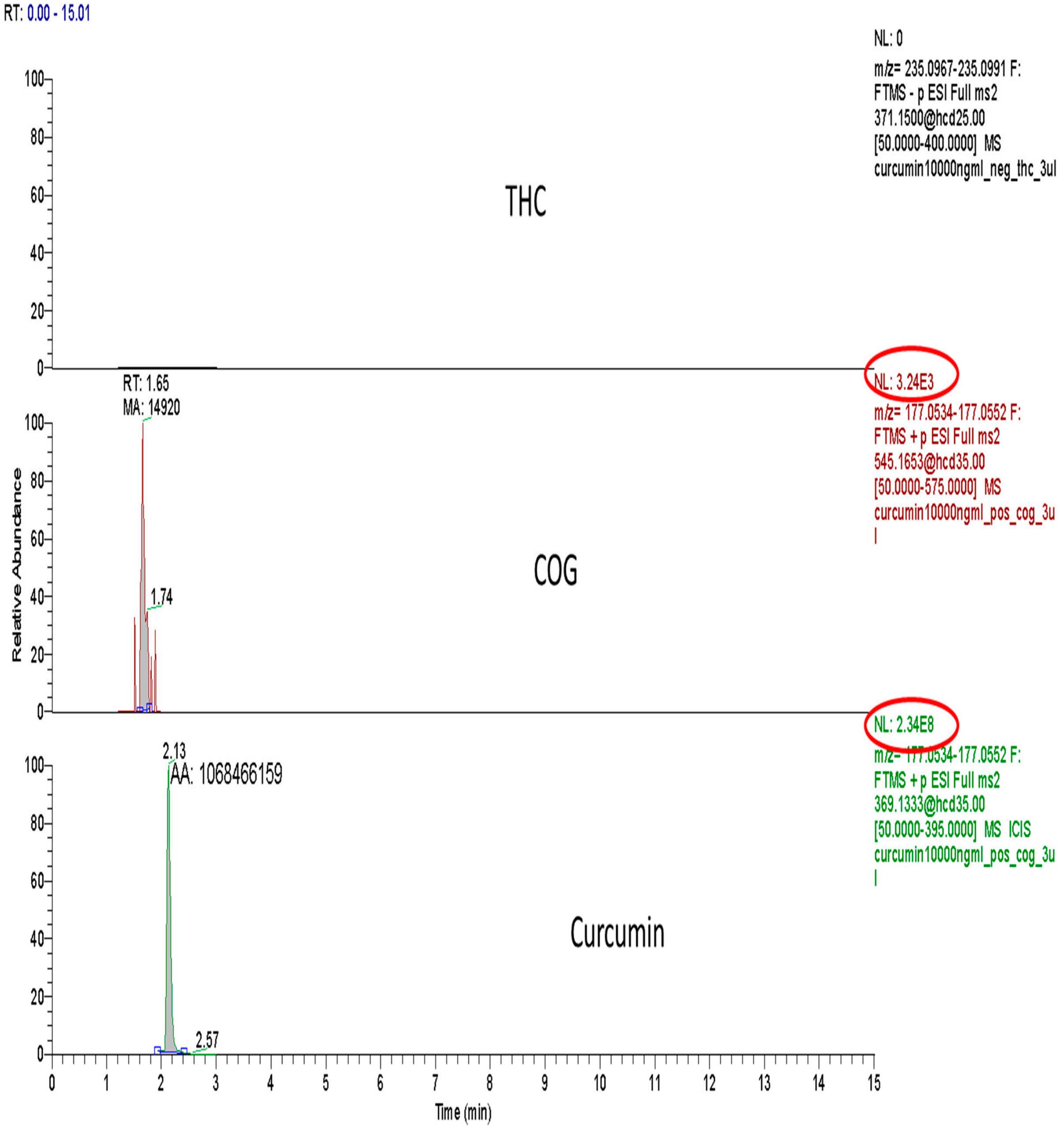

Supplementary Figure 2. Extracted ion chromatograms of THC, COG and Curcumin from the sample of curcumin powder, by LC-MS/MS. THC was not detected; COG was detected with a retention time of 1.65 min; Curcumin was detected with a retention time at 2.13 min.

Anti-Inflammatory Mechanisms of Curcumin in White Adipose Tissue of Diet-Induced Obese Male Mice

Islam et al, Nutrients.

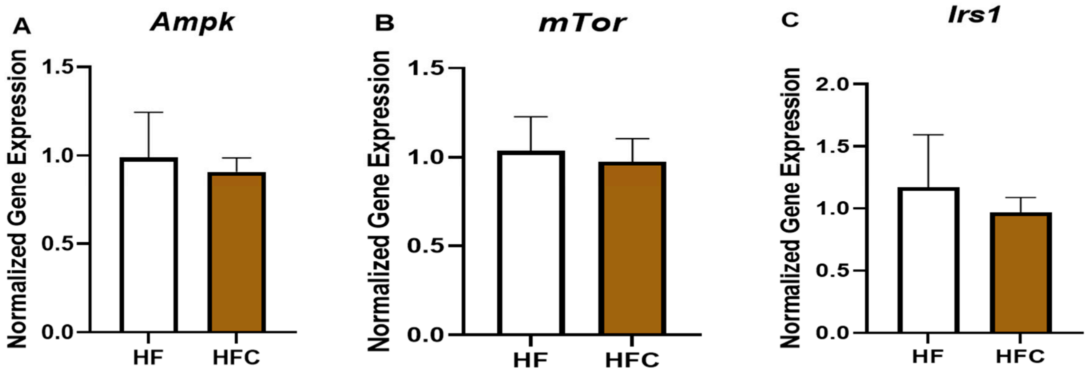

Supplementary Figure 3. Curcumin did not influence insulin signaling related gene expression in mice WAT. Insulin signaling-related genes (A) Ampk (B) mTOR and (C) Irs1 gene expression were not altered in WAT of HF vs HFC fed mice. Data is presented as ± SME (*N* = 6-9)
